# Fabrication of Customized Sectional Impression Trays in Management of Patients with Limited Mouth Opening: A Simple and Unique Approach

**DOI:** 10.1155/2013/275047

**Published:** 2013-07-24

**Authors:** Vamsi Krishna CH, K. Mahendranadh Reddy, Nidhi Gupta, Y. Mahadev Shastry, N. Chandra Sekhar, Venkat Aditya, G. V. K. Mohan Reddy

**Affiliations:** Department of Prosthodontics, Sri Sai College of Dental Surgery, Kothrepally, Vikarabad 501101, India

## Abstract

Impression making is not only important but is also the most significant step in the fabrication of any fixed or removable prosthesis. Proper impression making may be hindered by certain pathologic conditions. Reduced mouth opening is one of the common mechanical obstructions for proper orientation of the impression tray in the patient's mouth. In patients with trismus induced by submucous fibrosis, the procedure may be even more difficult to carry out because of reduced tissue resiliency and obliteration of vestibular spaces. Use of sectional trays offers one of the alternatives to overcome the problem of restricted mouth opening. Fabrication of customized impression trays according to the patient dentition improves the accuracy of impression making. The present case reports describe the fabrication of sectional custom trays designed for dentulous patients with chronic tobacco-induced submucous fibrosis.

## 1. Introduction

Reduced mouth opening poses a challenge and is often a daunting task for the operator to perform any intraoral procedures. Reportedly, this problem has been associated commonly with orofacial cancer surgeries, scleroderma, traumatic injuries, temporomandibular joint disorders, oral submucous fibrosis, and so forth. One of the most commonly observed pathologies associated with limited mouth opening is oral submucous fibrosis. Rajendran, in 1994 [1], reported and named this condition as “atrophia idiopathica (tropica) mucosae oris” involving oral mucosa, palate, and pillars of the fauces. Later, it was termed as oral sub mucous fibrosis. It is called by various synonyms like “diffuse oral sub mucous fibrosis,” “idiopathic scleroderma of the mouth,” “idiopathic palatal fibrosis,” “sclerosing stomatitis,” and “juxta-epithelial fibrosis” [2]. The characteristic finding observed in these patients is pale mucosa with loss of elasticity and resiliency. Formation of fibrous bands in sub mucous connective tissue was reported to be the root cause behind gradual reduction in mouth opening. Prosthetic intervention for these patients entails an accurate impression of the patient's mouth. Difficulties in impression making encountered due to reduced access to the oral cavity can be overcome by the use of sectional trays. Various types of sectional trays held together by different mechanisms have been designed and described in the literature. Present case reports describe simple and economic methods of fabrication of two-piece custom sectional trays for patients with oral sub mucous fibrosis.

## 2. Case Reports

**Figure 1:**
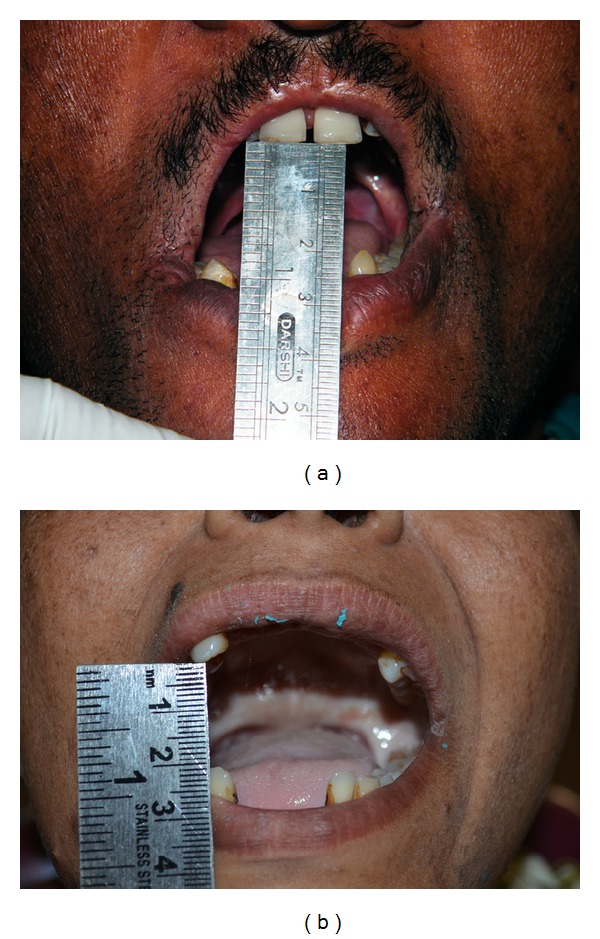
Maximum mouth openings.

45-year-old male patient and 31-year-old female patient, who were suffering from chronic oral sub mucous fibrosis, were reported to the department of prosthodontics (Sri Sai college of dental surgery, India) with a chief complaint of a missing teeth. On oral examination, maximum mouth opening was reported to be 2 cm and 2.4 cm, respectively, between incisal edges of maxillary and mandibular anteriors (Figure 1). Prognosis and probable prosthetic treatment options were explained to the patients, and informed consents were obtained.

Because of restricted size of oral orifice and severe intraoral fibrous bands, preliminary impressions were made with polyvinyl siloxane putty material. Flexible impression tray technique described by Whitsitt and Battle [3] was used to make preliminary impressions. The material was manipulated, rolled, and adapted on to the hard and soft tissues. Catalyst proportion was altered to reduce setting time to 1 min. Once the material had been set, the impression was folded and removed from the patient's mouth. The flexible impressions were stabilized using plaster and models obtained using pumice plaster method. Two-piece custom trays were designed and fabricated on the models.

### 2.1. Fabrication of Custom Trays

**Figure 2:**
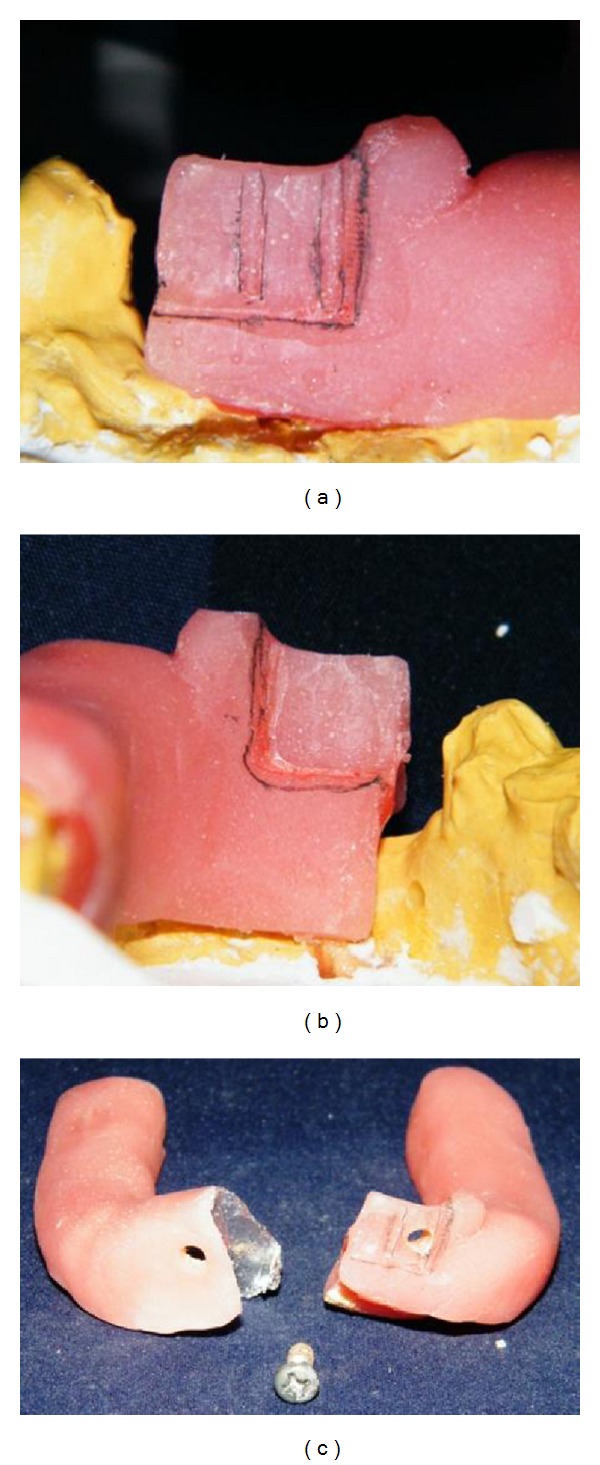
Fabrication technique for the sectional tray—Design 1.

*Design 1*. The custom tray was designed making sure that the sections of the tray could be joined firmly and oriented accurately both in patient's mouth and after removal of the tray from the mouth. A 2 mm thick wax spacer was adapted with four occlusal stops. Autopolymerizing resin was mixed and adapted using finger adaptation dough method on one side on the cast crossing midline. After the material polymerized, tray section was removed, trimmed, and designed using an acrylic trimmer as shown in Figure 2. The orientation grooves helped in three-dimensional stabilization of the tray. After designing the lock system for the first tray segment, a tin foil was adapted over that, and the second tray section was fabricated. After fabrication of the second segment, both the sections were approximated and secured using a screw. The screw helped in securing the tray segments together in a predetermined relationship (Figure 2).

**Figure 3:**
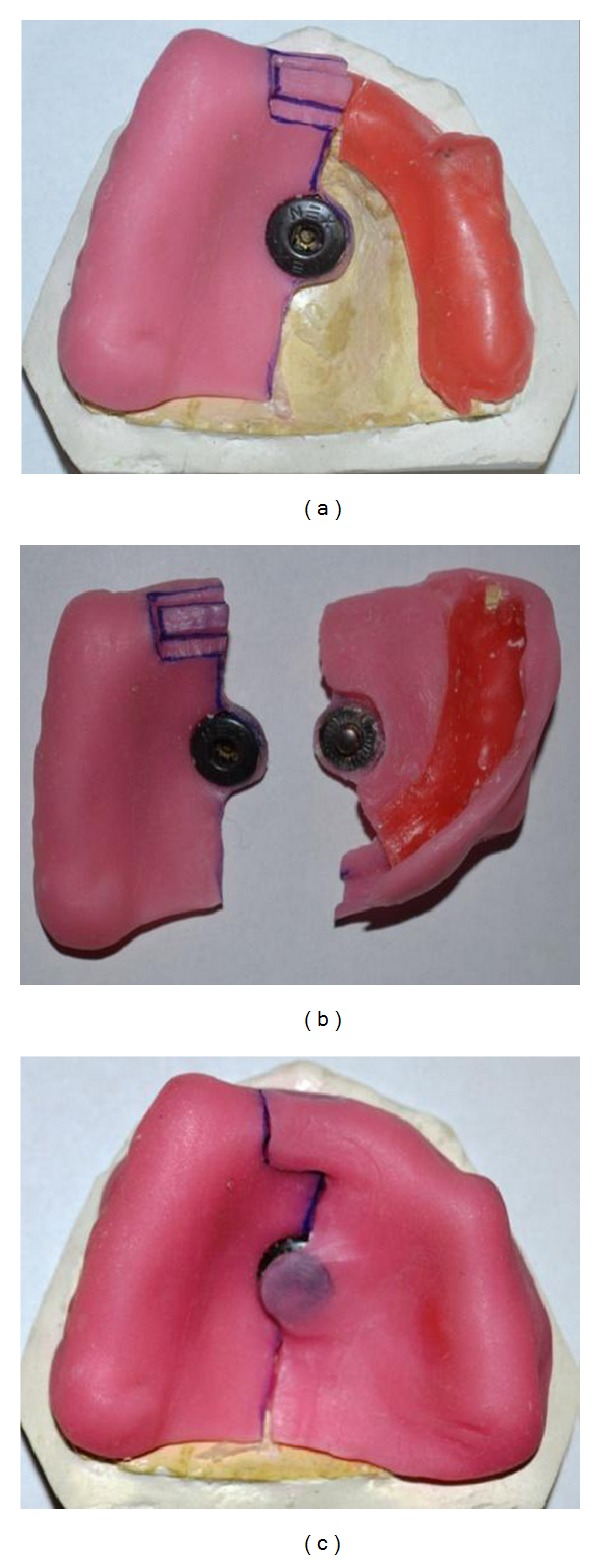
Fabrication technique for the sectional tray—Design 2.

*Design 2.* Fabrication of maxillary sectional tray was demonstrated here. After proper relief and block out, wax spacer was adapted on to the model. The first section of the tray was fabricated by adapting self-activated resin and incorporating female compartment of the press button on the center of the tray. Orientation lock was designed on the handle using acrylic trimmer as shown in Figure 3. After adapting tin foil separating medium, male part of the button was attached to female part, and the second segment tray was fabricated. During fabrication of the second segment, acrylic material was extended on to the orientation lock on the first segment near the handle of the tray. The female part of the button was retrieved along with the second section of the tray (Figure 3).

### 2.2. Impression Making

After completion of the special tray fabrication, the first segment was used to make the first section of the impression. Wax spacer was removed, and the tray was loaded with polyvinyl siloxane, and sectional impression was made. Sectional impression was removed, and the excess material flown on to the lock region and the screw hole was removed. The impression was placed back in the patient's mouth. The second part of the tray was loaded with the same impression material and oriented onto the first segment. 

**Figure 4:**
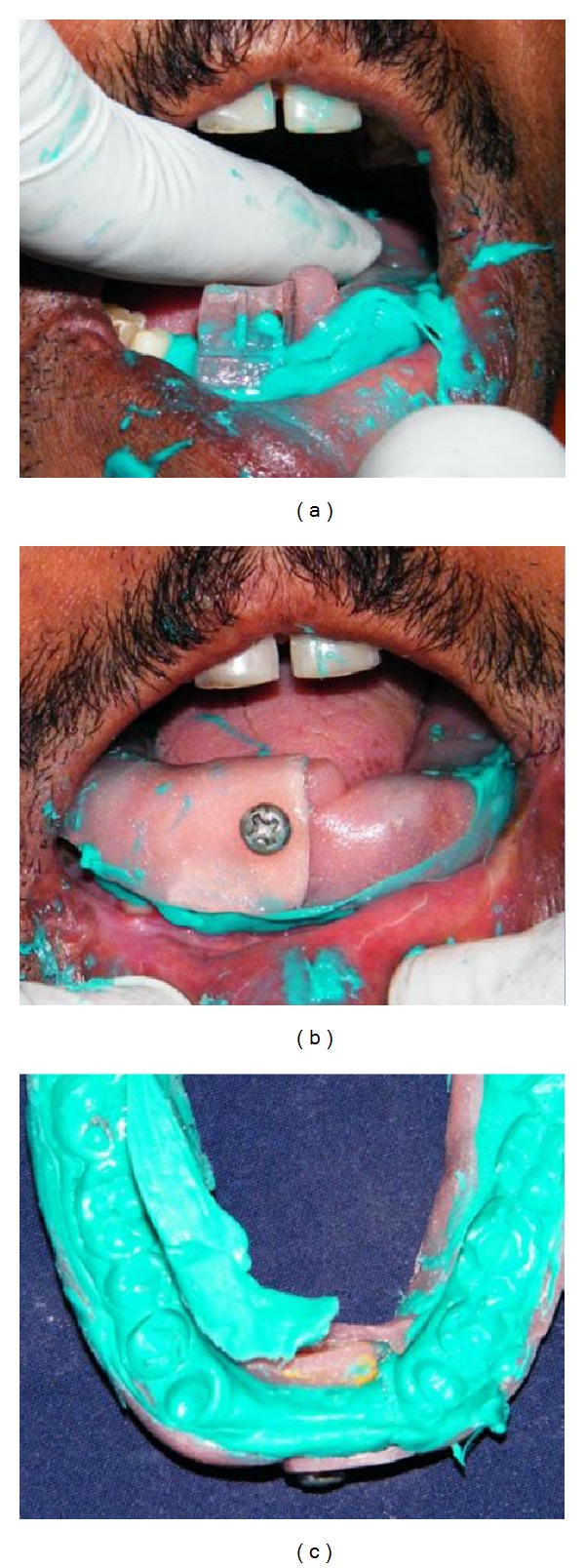
Impression making using the sectional tray Design 1.

In the first case report, after proper orientation of the tray, a screw was used to secure the segments together before the material set. The screw helped in securing the orientation of the sections of the tray properly within the patient's mouth. After the material had beenset, the screw was removed, and the sections were removed separately. Both the sections were approximated and secured using the screw after removal from the mouth (Figure 4). 

**Figure 5:**
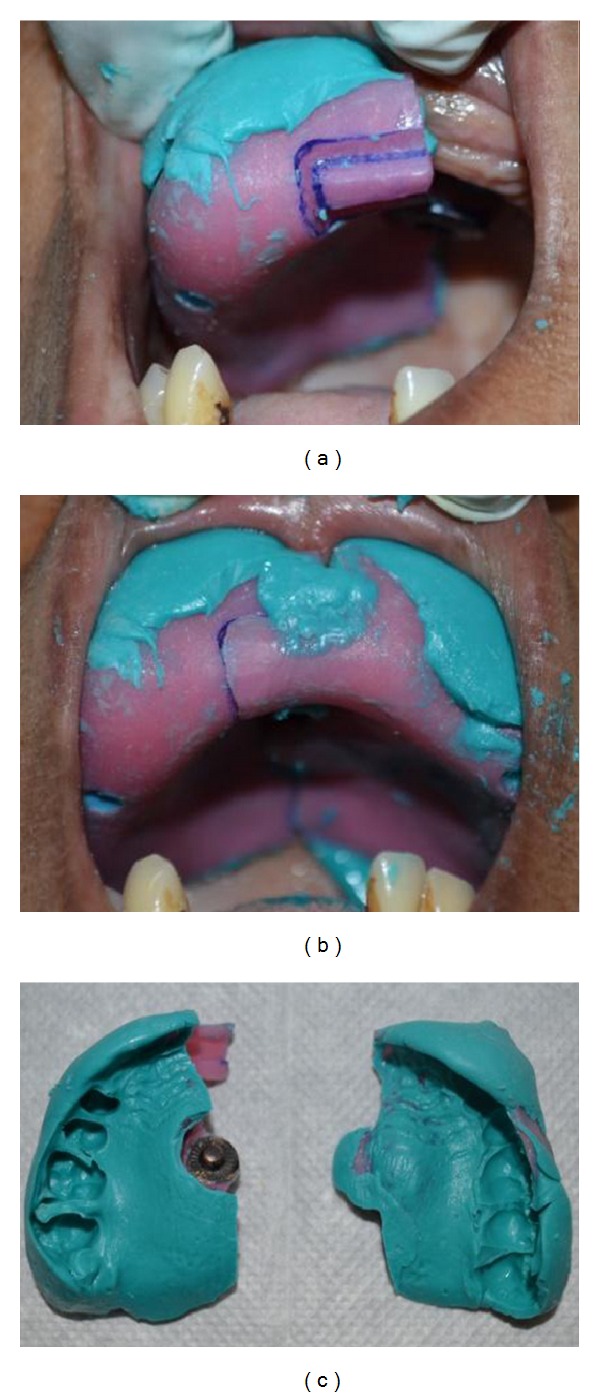
Impression making using the sectional tray Design 2.

In the second case report, the sections of the tray were oriented making sure that the male part of the button was seated properly onto the female part. After the material had been set, both the sections were separated and removed from mouth. Both the sections of the tray were joined together with the help of the locking button (Figure 5).

## 3. Discussion

Impression making in patients that planned for fixed or removable partial denture with restricted mouth opening is a challenging task as it requires more accuracy and precision. The present case reports described simplified locking designs of the tray segments which could be used for both dentulous and edentulous patients for fabrication of custom trays. 

In case report I, the patient was planned to receive a fixed partial denture replacing missing mandibular incisors. Patient in case report II was planned to receive a flexible removable partial denture to replace multiple missing teeth. Recording abutment finish line along with the remaining teeth is important for fabricating fixed partial denture. Similarly for fabrication of removable partial denture, the teeth along with functional depth of the sulcus have to be recorded. Practical difficulties of reduced mouth opening were overcome by designing sectional custom tray which provided an alternative for making an accurate impression.

Simple and economic sectional tray design was fallowed in the present case reports. Male and female segments of the tray were oriented by the locking mechanism which was designed using acrylic trimmer. Use of screws and press buttons helped in securing tray segments more accurately together with precession. Many techniques were described in the literature for impression making in dentulous and edentulous patients with limited mouth opening. Various mechanisms like hinges [4], locking levers [5], plastic blocks [6, 7], orthodontic expansion screws [8], magnet systems [9], parallel pins [10], and so forth were used so far for fabricating sectional trays. In the present case reports, incorporation of complicated locking devices was avoided by designing a locking mechanism within the tray handle to secure the tray segments three dimensionally. Manual locks designed with trimmer on the surface of acrylic lack accuracy and precession. Incorporation of screw and press button into the design provides the precession in securing the trays together firmly. Accurate fit of fixed and removable prostheses was reported with the impressions obtained from both sectional tray designs.

## 4. Conclusions

Simple alterations in procedural techniques help to overcome clinical difficulties faced during prosthetic management of patients with oral sub mucous fibrosis. Present case reports facilitated the operator to obtain accurate impressions for patients with limited mouth opening. These simple and logical sectional tray designs are easy to fabricate, consume less time, and require inexpensive locking mechanisms.
